# Phase I study of lurbinectedin in combination with weekly paclitaxel with or without bevacizumab in patients with advanced solid tumors

**DOI:** 10.1007/s10637-022-01281-z

**Published:** 2022-08-10

**Authors:** Emiliano Calvo, Cristiana Sessa, Guilherme Harada, Maria de Miguel, Carmen Kahatt, Xarles Erik Luepke-Estefan, Mariano Siguero, Carlos Fernandez-Teruel, Martin Cullell-Young, Anastasios Stathis, Alexander Drilon

**Affiliations:** 1 START Madrid - HM CIOCC, Hospital Madrid Norte Sanchinarro, Madrid, Spain; 2 Oncology Institute of Southern Switzerland, EOC, Ospedale San Giovanni, Bellinzona, Switzerland; 3 Memorial Sloan Kettering Cancer Center and Weill Cornell Medical College, New York, NY USA; 4 PharmaMar, Colmenar Viejo, Madrid, Spain

**Keywords:** Lurbinectedin, Paclitaxel, Bevacizumab, Phase I study

## Abstract

Lurbinectedin and paclitaxel showed synergism in preclinical studies and have non-completely overlapping toxicity profiles. This phase I trial evaluated a combination of paclitaxel and lurbinectedin with/without bevacizumab in advanced tumors. This trial was divided into Group A, which evaluated weekly paclitaxel (60 or 80 mg) plus lurbinectedin (3.0–5.0 mg flat dose [FD] or 2.2 mg/m^2^) every 3 weeks in advanced solid tumors; and Group B, which evaluated bevacizumab (BEV, 15 mg/kg) added to the recommended dose (RD) defined in Group A in advanced epithelial ovarian or non-small cell lung cancer (NSCLC). 67 patients (A, n = 55; B, n = 12) were treated. The RD was paclitaxel 80 mg/m^2^ on Day (D)1,D8 plus lurbinectedin 2.2 mg/m^2^ on D1. At this RD, myelotoxicity was reversible and manageable, and most non-hematological toxicities were mild/moderate. Adding BEV did not notably change tolerability. Twenty-five confirmed responses were observed: 20/51 evaluable patients in Group A (overall response rate [ORR] = 39% at all dose levels and at the RD), and 5/10 evaluable patients in Group B (ORR = 50%). Most responders had breast (n = 7/12 patients), small cell lung (SCLC) (n = 5/7), epithelial ovarian (n = 3/9) and endometrial cancer (n = 3/11) in Group A, and epithelial ovarian (n = 3/4) and NSCLC (n = 2/6) in Group B. Clinical benefit rate was 61% in Group A (58% at the RD), and 90% in Group B. No major pharmacokinetic drug-drug interactions were observed. Paclitaxel/lurbinectedin and paclitaxel/lurbinectedin/BEV are feasible combinations. Further development is warranted of paclitaxel/lurbinectedin in SCLC, breast, and endometrial cancer, and of paclitaxel/lurbinectedin/BEV in epithelial ovarian cancer.

**Supplementary Information:**

The online version contains supplementary material available at 10.1007/s10637-022-01281-z.

## Introduction

Lurbinectedin (Zepzelca^®^) is a synthetic tetrahydroisoquinoline alkaloid structurally related to trabectedin. It inhibits oncogenic transcription primarily through binding to the exocyclic amino group of guanine-rich DNA sequences around promoters of protein-coding genes, thereby altering the 3D DNA structure and evicting oncogenic transcription factors from their binding sites, thus halting their aberrant transcription programs [1–3]. Lurbinectedin adducts can stop transcribing (phosphorylated) RNA polymerase II, decreasing mRNA synthesis and inducing the ubiquitination and degradation of RNA polymerase II inhibition [4]. Lurbinectedin adducts may also trick the nucleotide excision repair system, favoring the production of DNA double-strand breaks and triggering apoptotic cell death [5]. Lurbinectedin monotherapy has been approved in the U.S. and other countries for the treatment of adult patients with metastatic small cell lung cancer (SCLC) and disease progression on or after platinum-based chemotherapy.

The first-in-human phase I study defined a recommended dose (RD) of 7 mg flat dose (FD) for single-agent lurbinectedin as a 1-h intravenous (i.v.) every 3 weeks (q3wk) [6]. Severe but transient neutropenia, and mild fatigue, nausea and vomiting, were common at this RD. The pharmacokinetic (PK) profile of lurbinectedin showed dose linearity, high interpatient variability, and a long median half-life (70.6 h at the RD).

Preclinical studies showed improved antitumor activity for lurbinectedin with taxanes. In vivo, synergism was observed with lurbinectedin and paclitaxel in mice bearing gastric, ovarian, non-small cell lung cancer (NSCLC), breast or prostate xenografted tumors [7]. Lurbinectedin and paclitaxel have toxicity profiles that are not completely overlapping. Both are cytochrome CYP3A4 substrates; hence, PK interactions by competitive inhibition of this enzyme system cannot be discarded.

Bevacizumab (BEV) is a humanized monoclonal antibody against the circulating vascular endothelial growth factor that inhibits tumor angiogenesis. BEV primarily acts in the tumor microenviroment, with very little hematological toxicity. The combination of chemotherapy with BEV has been associated with improved clinical activity compared with chemotherapy alone [8–13].

The aim of this phase I study was to determine the maximum tolerated dose (MTD) and the RD, safety profile, activity and PK of lurbinectedin combined with weekly paclitaxel, with or without BEV, in advanced solid tumor patients.

## Patients and methods

Supplementary Information includes details regarding study design and eligibility criteria. In brief, patient accrual began in Group A (paclitaxel and lurbinectedin) at a starting dose of paclitaxel 60 mg/m^2^ plus lurbinectedin 3.0 mg flat dose (FD). Treatment initially consisted of escalating doses of paclitaxel as 1-h i.v. infusions on Day (D) 1, D8 and D15, followed by lurbinectedin as a 1-h i.v. infusion on D1, both every three weeks (q3wk). After DL3, the paclitaxel schedule was changed to 1-h i.v. infusions on D1 and D8 q3wk owing to a high incidence of D15 dose omissions in DL3. Furthermore, during dose escalation lurbinectedin was converted to a body surface area (BSA)-based dose. Once the RD for paclitaxel and lurbinectedin had been determined in Group A, patients were enrolled in Group B and received this RD supplemented with BEV 15 mg/kg as a 30–90 min i.v. infusion on D1 q3wk.

In both groups, paclitaxel was discontinued after Cycle 6; patients still on treatment continued receiving lurbinectedin alone (Group A) or lurbinectedin plus BEV (Group B) at the same dose. Treatment was administered until disease progression, unacceptable toxicity, intercurrent illness precluding study continuation, patient refusal and/or non-compliance with study requirements, treatment delay > 15 days (except if with clear clinical benefit), and > 2 dose reductions.

### Study assessments

Supplementary Information includes definitions for dose-limiting toxicities (DLTs) and details regarding safety, efficacy and PK assessments.

Adverse events (AEs) and laboratory abnormalities were graded with the National Cancer Institute Common Terminology Criteria for Adverse Events (NCI-CTCAE) v.4 [14], and coded using the Medical Dictionary for Regulatory Activities (MedDRA) v.16.0. Antitumor activity was evaluated every three cycles according to the RECIST v.1.1 [15]. The PK analysis was conducted on 12 blood samples collected from each patient during Cycle 1 to quantify lurbinectedin and paclitaxel plasma concentrations.

### Statistical analysis

Continuous variables were presented with summary statistics and categorical variables in frequency tables. Time-to-event variables were calculated using Kaplan–Meier approach. Binomial exact distribution was used to calculate 95% confidence intervals (95%CIs) for categorical variables. Blood and plasma concentration–time profiles were analyzed by standard non-compartmental methods. Individual PK parameters were tabulated and summarized.

## Results

### Patient characteristics

Sixty-nine patients were enrolled in this study: 55 in Group A (paclitaxel/lurbinectedin) and 14 in Group B (paclitaxel/lurbinectedin/BEV). Patient characteristics at baseline are summarized in Table 1.

**Table 1:** Baseline characteristics of patients

	**Group A** **(Paclitaxel / lurbinectedin)**		**Group B** **(Paclitaxel / lurbinectedin / BEV)**
	**RD** (n = 37)	**All dose levels** (n = 55)	**RD + BEV** (n = 14)
**Gender**			
Female	26 (70%)	40 (73%)	8 (57%)
Male	11 (30%)	15 (27%)	6 (43%)
**Median age, years (range)**	61 (38–74)	57 (31–74)	57 (47–72)
**ECOG performance status**			
0	22 (59%)	33 (60%)	11 (79%)
1	15 (41%)	22 (40%)	3 (21%)
**Median BSA, m**^**2**^** (range)**	1.8 (1.4–2.2)	1.8 (1.4–2.4)	1.8 (1.4–2.2)
**Primary tumors**			
Breast	8 (22%)	12 (22%)	
Endometrial	13 (35%)	13 (24%)	
Epithelial ovarian	3 (8%)	9 (16%)	7 (50%)
NSCLC	6 (16%)	9 (16%)	7 (50%)
SCLC	5 (14%)	7 (13%)	
Other ^a^	2 (5%)	5 (9%)	
**Bulky disease (any target lesion ≥ 50 mm)**	5 (14%)	8 (15%)	1 (7%)
**Median number of metastatic sites (range)**	2 (1–6)	2 (1–6)	3 (1–6)
**Most common sites of disease**			
Liver	15 (41%)	24 (44%)	4 (29%)
Lung	15 (41%)	24 (44%)	8 (57%)
Lymph node	23 (62%)	33 (60%)	7 (50%)
Peritoneum	12 (32%)	19 (35%)	6 (43%)
**Prior treatment for advanced disease**			
Median (range)	1 (0–4)	2 (0–7)	2 (0–4)
0 ^b^	1 (3%)	1 (2%)	1 (7%)
1	20 (54%)	25 (45%)	5 (36%)
2	8 (22%)	14 (25%)	3 (21%)
3 or more	8 (22%)	15 (27%)	5 (36%)
**Prior anticancer agents **^**c**^			
Anthracyclines and related substances	16 (43%)	24 (44%)	4 (33%)
Aromatase inhibitors	3 (8%)	8 (15%)	
Folic acid analogues	4 (11%)	7 (13%)	7 (58%)
Monoclonal antibodies	5 (14%)	11 (20%)	4 (33%)
Nitrogen mustard analogues	12 (32%)	15 (27%)	
Platinum compounds	32 (87%)	45 (82%)	12 (100%)
Podophyllotoxin derivatives	6 (16%)	8 (15%)	1 (8%)
Pyrimidine analogues	13 (35%)	20 (36%)	5 (42%)
Taxanes	24 (65%)	33 (60%)	8 (67%)

Data shown are number of patients (percentage), except for median (range)

^a^ Includes adenocarcinoma or carcinoma of unknown primary site (n = 2), cervical cancer (n = 1), platinum-refractory GCTs (n = 1), and HNSCC (n = 1)

^b^ These patients received prior therapy in the adjuvant setting

^c^ Calculated on the number of patients treated (n = 55 at all dose levels in Group A, n = 37 at the RD in Group A, and n = 12 in Group B)

*BEV* bevacizumab, *BSA* body surface area, *CNS* central nervous system, *ECOG* Eastern Cooperative Oncology Group, *GCT* germ cell tumor, *HNSCC* head and neck squamous cell carcinoma, *NSCLC* non-small cell lung cancer, *RD* recommended dose, *SCLC* small cell lung cancer

Most of the 55 patients (73%) enrolled at all dose levels in Group A were female, with median age 57 years (range, 31–74 years). The most common primary tumors comprised endometrial (n = 13 patients, 24%), breast (n = 12, 22%), epithelial ovarian, NSCLC (n = 9, 16% each) and SCLC (n = 7, 13%). The median number of metastatic sites was 2 (range, 1–6 sites). The median number of lines of all prior therapies for advanced disease was 2 (range, 0–7 lines), and the most common prior therapies were platinum compounds (82%) and taxanes (60%). Thirty-seven patients in Group A were enrolled at the RD. Twenty-six of these patients (70%) were female, with a median age of 61 years (range, 38–74 years). Their most common primary tumors were endometrial (35% of patients), breast (22%), NSCLC (16%), SCLC (14%) and epithelial ovarian (8%). The median number of metastatic sites was 2 (range, 1–6 sites) and the median number of lines of prior therapy for advanced disease was 1 (range, 0–5 lines), with the most common prior therapies being platinum compounds (87%) and taxanes (65%).

Most of the 14 patients (57%) enrolled in Group B were also female. The median age was 57 years (range, 47–72 years). All patients had epithelial ovarian cancer or NSCLC (n = 7 patients each). The median number of metastatic sites was 3 (range, 1–6 sites), and the median number of lines of prior therapy for advanced disease was 2 (range, 0–4 lines). All 14 patients had been pre-treated with platinum compounds; other prior therapies were taxanes (67%) and folic acid analogues (58%).

### Treatment administration

In Group A, 55 enrolled patients were treated at five dose levels: DL1 (n = 3 patients), DL2 (n = 3) and DL3 (n = 6) with paclitaxel on D1, D8 and D15 q3wk, and DL4 (n = 6) and DL5 (n = 37) with paclitaxel on D1 and D8 q3wk (see Supplementary Information). A total of 392 treatment cycles were administered at all dose levels (median: 5.0 cycles per patient); 20 patients (36%) were still on treatment after Cycle 6 and switched to single-agent lurbinectedin. At DL5 (the RD), 256 treatment cycles were given (median: 5.0 cycles per patient) and 15 patients (41%) switched to single-agent lurbinectedin after Cycle 6. Most treatment discontinuations (44 of 55 patients [80%] at all dose levels; 30 of 37 patients [81%] at DL5 [the RD]) were due to disease progression. No treatment-related discontinuations occurred at the RD. The median time on treatment was 4.8 months, both at all dose levels and at the RD. The median dose intensities (DIs) at the RD were 49.1 mg/m^2^/week for paclitaxel and 0.7 mg/m^2^/week for lurbinectedin, and the median relative DIs compared to the initially planned dose were 92% and 98%, respectively. At the RD, 6 patients (16%) had cycle delays and 7 patients (19%) had study drug dose reductions due to treatment-related reasons (mostly hematological toxicity).

In Group B, 12 of the 14 enrolled patients received a total of 111 treatment cycles at a dose of paclitaxel 80 mg/m^2^ on D1 and D8 and lurbinectedin 2.2 mg/m^2^ on D1 q3wk supplemented with BEV 15 mg/kg on D1 q3wk (median: 9.5 cycles per patient). Two patients were withdrawn from the study before receiving the first dose due to disease-related bowel obstruction (n = 1), and patient refusal (n = 1). Eight patients (67%) were still on treatment after Cycle 6 and switched to lurbinectedin and BEV. Most treatment discontinuations (8 of 12 patients [67%]) were due to disease progression. One discontinuation was due to treatment-related thrombocytopenia. The median time on treatment was 7.9 months. The median DIs were 50.3 mg/m^2^/week for paclitaxel, 0.7 mg/m^2^/week for lurbinectedin and 5.0 mg/m^2^/week for BEV; the median relative DIs were 94%, 100% and 99%, respectively. Three patients (25%) had cycle delays and 2 patients (17%) had study drug dose reductions owing to treatment-related reasons (mostly hematological toxicity).

### Dose escalation and recommended dose

Fifty-two treated patients in Group A were evaluable for DLTs (see Supplemental information). Three patients were non-evaluable because they did not receive a complete Cycle 1 due to disease-related events (n = 2), or because of lack of laboratory assessment for DLT evaluation during Cycle 1 (n = 1). Paclitaxel was initially administered at a schedule of D1, D8 and D15 q3wk. No DLTs occurred at the first two dose levels. Two of 6 patients at DL3 (paclitaxel 60 mg/m^2^, lurbinectedin 5.0 mg FD) had delayed DLTs (grade 3 or 4 neutropenia after Cycle 1). After DL3, the paclitaxel schedule was changed to D1 and D8 q3wk owing to the finding of a high incidence (4 of 6 patients) of paclitaxel dose omissions due to treatment-related neutropenia during Cycle 1 at DL3. At DL4 (paclitaxel 80 mg/m^2^, lurbinectedin 5.0 mg FD), 3 of 6 patients had DLTs (grade 3/4 neutropenia > 7 days [n = 2]; or lack of compliance due to neutropenia [n = 1]); as a result, this dose was defined as the MTD. None of the first 6 patients at DL5 (paclitaxel 80 mg/m^2^, lurbinectedin 4.0 mg FD) had DLTs. The DL5 cohort was then expanded, converting lurbinectedin to a BSA-based dose of 2.2 mg/m^2^ (calculated by dividing 4.0 mg FD by a BSA of 1.8 m^2^). Six of 28 patients in the expanded DL5 cohort (paclitaxel 80 mg/m^2^, lurbinectedin 2.2 mg/m^2^) had DLTs (grade 3 neutropenia > 7 days alone [n = 2] or with grade 3 anemia [n = 1], grade 4 neutropenia > 3 days [n = 1], grade 2 anemia and grade 2 tooth infection (with concomitant neutropenia) [n = 1], and grade 3 vomiting [n = 1]), thereby confirming paclitaxel 80 mg/m^2^ on D1 and D8 plus lurbinectedin 2.2 mg/m^2^ on D1 as the RD in Group A, with prolonged severe neutropenia as the most common DLT. No episodes of febrile neutropenia occurred as DLTs in Group A.

All 12 patients treated with paclitaxel 80 mg/m^2^ on D1 and D8 plus lurbinectedin 2.2 mg/m^2^ on D1 and BEV 15 mg/kg on D1 in Group B were DLT evaluable (see Supplemental information). Three patients had DLTs: grade 3 febrile neutropenia (n = 1), grade 4 neutropenia > 3 days (n = 1), and grade 4 large intestine perforation (n = 1, a patient with ovarian cancer). No treatment-related deaths occurred in Group B. The percentage of patients with DLTs in Group B (3/12 patients; 25%) in the absence of treatment-related mortality was below the threshold of one third of patients defined in the study protocol, thereby confirming the feasibility of adding BEV to the RD defined in Group A.

### Safety

All treated patients were evaluable for safety. Treatment-related AEs and laboratory abnormalities at the RD in Group A and in Group B are shown in Table 2.

**Table 2:** Treatment-related adverse events (> 10% of patients) and laboratory abnormalities (hematological and biochemical) at the recommended dose for phase II studies, with or without BEV

	**Group A** (**Paclitaxel 80 mg/m**^**2**^** / lurbinectedin 4.0 mg FD or 2.2 mg/m**^**2**^**)** (n = 37)					**Group B** **(Paclitaxel 80 mg/m**^**2**^** / lurbinectedin 2.2 mg/m**^**2**^** / BEV 15 mg/kg)** (n = 12)			
**NCI-CTCAE grade**	**1–2**	**3**	**4**	**Total**		**1–2**	**3**	**4**	**Total**
**Hematological laboratory abnormalities**									
**Anemia **^**a**^	72	22		94		83	17		100
**Neutropenia **^**a**^	14	31	28	72			42	25	67
**Thrombocytopenia **^**a**^	31	17		47		25	17		42
**Biochemical laboratory abnormalities**									
**ALT increased **^**a**^	47	11	3	61		75			75
**AP increased **^**a**^	36	6		42		42			42
**AST increased **^**a**^	44	6	3	53		42			42
**Bilirubin increased **^**a**^	11	3		14					
**CPK increased **^**a**^	11			11		17			17
**Creatinine increased **^**a**^	81			81		75			75
**Adverse events**									
**Abdominal abscess**							17		17
**Alopecia**	24			24		42			42
**Constipation**	8			8		25			25
**Decreased appetite**	16			16		25			25
**Diarrhea**	22	3		24		33			33
**Dysgeusia**	11			11					
**Dyspepsia**	11			11		17			17
**Epistaxis**						17			17
**Fatigue**	57	3		59		50	8		58
**Febrile neutropenia**		3		3			17		17
**Hypertension**						25			25
**Nausea**	51	3		54		50			50
**Peripheral sensory neuropathy**	27			27		17			17
**Pulmonary embolism**							17		17
**Rash maculo-papular**	3	3		5		17			17
**Vomiting**	43	3		46		8			8

The percentage of patients with each adverse event is specified

Hematological and biochemical abnormalities are shown regardless of relationship to treatment

^a^ Missing data for one patient in Group A

*ALT* alanine aminotransferase, *AP* alkaline phosphatase, *AST* aspartate aminotransferase, *BEV* bevacizumab, *CPK* creatine phosphokinase, *FD* flat dose, *NCI-CTCAE* National Cancer Institute Common Terminology Criteria for Adverse Events

Thirty-four of 37 patients (92%) treated at the RD in Group A had at least one treatment-related AE. Most of these AEs were grade 1/2, with the most common being fatigue (59% of patients), nausea (54%), vomiting (46%), peripheral sensory neuropathy (27%), diarrhea and alopecia (24% each). Grade ≥ 3 treatment-related AEs comprised catheter site cellulitis, diarrhea, fatigue, maculopapular rash, nausea, sepsis, and vomiting in one patient each (3%). Of these, only sepsis reached grade 4. Hematological abnormalities consisted of anemia (94%; grade 3 in 22%), neutropenia (72%; grade 3/4 in 58%) and thrombocytopenia (47%; grade 3 in 17%); one patient (3%) had treatment-related grade 3 febrile neutropenia. The most frequent biochemical abnormalities were increases in creatinine (81%), alanine aminotransferase (ALT) (61%; grade 3/4 in 14%), aspartate aminotransferase (AST) (53%; grade 3/4 in 8%) and AP (42%, grade 3 in 6%). Seven patients (18.9%) required red blood cell (RBC) transfusions and four patients (10.8%) required secondary granulocyte colony-stimulating factor (G-CSF) support. No treatment discontinuations or deaths occurred due to toxicity.

All 12 treated patients in Group B had at least one treatment-related AE. Most of these AEs were also grade 1/2, with the most frequent being fatigue (58%), nausea (50%), alopecia (42%) and diarrhea (33%). Grade ≥ 3 treatment-related AEs comprised abdominal abscess and pulmonary embolism in two patients each (17%), and colonic fistula, fatigue, gastrointestinal fistula, large intestine perforation, and septic shock in one patient each (8%); of these, only the intestinal perforation was grade 4. Hematological abnormalities consisted of anemia (all patients; grade 3 in 17%), neutropenia (67%; all grade 3/4) and thrombocytopenia (42%; grade 3 in 17%). Two patients (17%) had grade 3 febrile neutropenia. All biochemical abnormalities were grade 1/2, with the most common being increases in creatinine and ALT (75% each), AST and AP (42% each). Three patients (25.0%) required RBC transfusions and three (25.0%) were given G-CSF support. One patient discontinued treatment due to toxicity (grade 4 large intestine perforation). No deaths occurred due to toxicity.

### Efficacy

Fifty-one patients in Group A and 10 patients in Group B were evaluable for efficacy. Four patients in Group A and 2 in Group B were not evaluable because no tumor evaluations were conducted after baseline imaging.

**Fig. 1:**
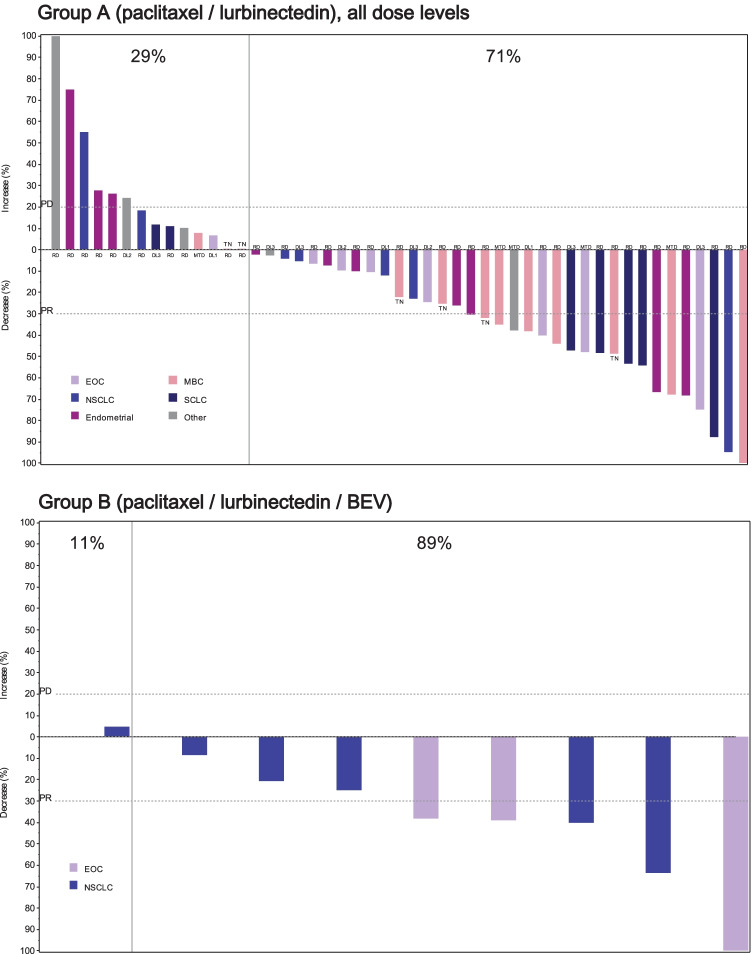
Maximum variation of target lesions in treated patients with measurable disease and at least one radiological tumor assessment at all dose levels in Group A (paclitaxel plus lurbinectedin) (n = 49), and in Group B (paclitaxel plus lurbinectedin and BEV) (n = 9). BEV, bevacizumab; DL, dose level; EOC, epithelial ovarian cancer; MBC, metastatic breast cancer; MTD, maximum tolerated dose; NSCLC, non-small cell lung cancer; PD, progressive disease; PR, partial response; RD, recommended dose; SCLC, small cell lung cancer; TN, triple negative

In Group A, 20 patients had confirmed responses, including one complete response (CR) (overall response rate [ORR] = 39%; 95%CI, 25.8–53.9%) and 11 patients had stable disease (SD) ≥ 3 months (clinical benefit rate [CBR] = 61%; 95%CI, 46.1–74.2%) at all dose levels, with objective tumor shrinkage in 35 (71%) of 49 evaluable patients (Fig. 1). The median progression-free survival (PFS) was 3.9 months (95%CI, 1.9–5.6 months) and the median DoR was 2.6 months (95%CI, 2.0–6.1 months). Thirteen confirmed responses (including one CR) and 6 SD ≥ 3 months occurred at the RD (ORR = 39%, 95%CI, 22.9–57.9%; CBR = 58%, 95%CI, 39.2–74.5%), with objective tumor shrinkage in 22 (69%) of 32 evaluable patients (Table 3 and Fig. 1). At the RD, the median PFS was 3.9 months (95%CI, 1.9–6.0 months) and the median DoR was 4.1 months (95%CI, 2.1–8.3 months). The highest ORRs were found in patients with SCLC (n = 5, 71% at all dose levels; n = 4, 80% at the RD), breast cancer (n = 7, 58%; and n = 4, 50%, respectively), epithelial ovarian (n = 3, 33%; and n = 1, 33%, respectively), endometrial cancer (n = 3, 27% at all dose levels and the RD), and NSCLC (n = 1, 14%; and n = 1, 25%, respectively).

**Table 3:** Antitumor activity according to RECIST in patients evaluable for efficacy at the recommended dose for phase II studies, with or without BEV

	**Group A** (**Paclitaxel 80 mg/m**^**2**^** / lurbinectedin 4.0 mg FD or 2.2 mg/m**^**2**^**)** (n = 33)		**Group B** **(Paclitaxel 80 mg/m**^**2**^** / lurbinectedin 2.2 mg/m**^**2**^** / BEV 15 mg/kg)** (n = 10)	
	**n**	**%**	**n**	**%**
**CR**	1	3	1	10
**PR**	12	36	4	40
**SD ≥ 3 mo**	6	18	4	40
**SD < 3 mo**	1	3		
**PD**	12	36		
**Early PD**	1	3		
**TF**			1	10
**ORR (%)** **(95%CI)**	39% (22.9–57.9%)		50% (18.7–81.3%)	
**CBR (%)** **(95%CI)**	58% (39.2–74.5%)		90% (55.5–99.8%)	
**Median DoR (months)** **(95%CI)**	4.1 (2.1–8.3)		4.6 (1.4-n.r.)	

*BEV* bevacizumab, *CBR* clinical benefit rate, *CI* confidence interval, *CR* complete response, *D* Day, *DL* dose level, *DoR* duration of response, *FD* flat dose, *MTD* maximum tolerated dose, *n.r*. not reached, *ORR* overall response rate, *PD* progressive disease, *PR* partial response, *q3wk* every three weeks, *RD* recommended dose, *RECIST* Response Evaluation Criteria In Solid Tumors, *SD* stable disease, *TF* treatment failure

In Group B, 5 patients had confirmed responses, including one CR (ORR = 50%; 95%CI, 18.7–81.3%) and 4 patients had SD ≥ 3 months (CBR = 90%; 95%CI, 55.5–99.8%) (Table 3). Objective tumor shrinkage was found in 8 (89%) of 9 patients with ≥ 1 radiological tumor assessment (Fig. 1). The median PFS was 6.7 months (95% CI, 2.4–9.3 months) and the median DoR was 4.6 months (95% CI, 1.4 months-not reached). Of the 5 patients with confirmed response, 3 had epithelial ovarian cancer (ORR = 75%) and 2 had NSCLC (ORR = 33%).

**Fig. 2:**
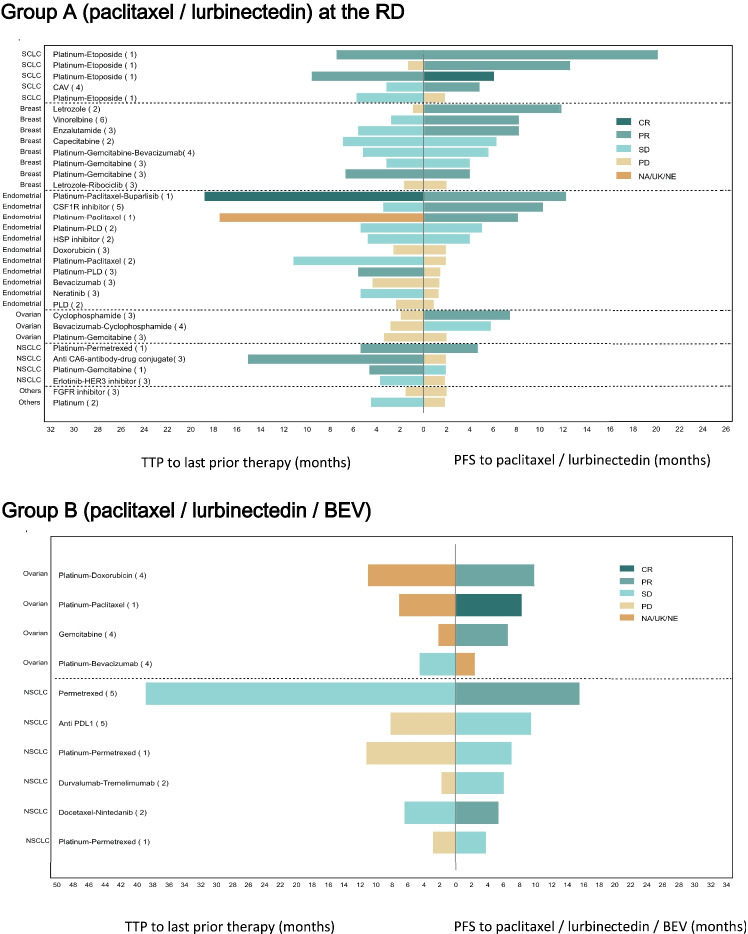
Swimmer plot of best response as per RECIST to study treatment *vs.* last prior therapy at the RD in Group A (paclitaxel plus lurbinectedin) (n = 33) and in Group B (paclitaxel plus lurbinectedin and BEV) (n = 10). The tumor type, last prior therapy, and total number of prior lines (in parenthesis) of each patient is shown at the left of the figure. BEV, bevacizumab; CAV, cyclophosphamide, doxorubicin and vincristine; CR, complete response; CSF1R, colony-stimulating factor-1 receptor; FGFR, fibroblast growth factor receptor; HSP, heat shock protein; NA, not available; NE, not evaluable; NSCLC, non-small cell lung cancer; PD, progressive disease; PDL1, programmed death ligand-1; PFS, progression-free survival; PLD, pegylated liposomal doxorubicin; PR, partial response; RD, recommended dose; RECIST, Response Evaluation Criteria In Solid Tumors; SCLC, small cell lung cancer; SD, stable disease; TTP, time to progression; UK, unknown

The best response to paclitaxel and lurbinectedin with or without BEV was compared with response to the last prior therapy in evaluable patients treated at the RD in Group A and in Group B. Overall, 9 of 33 patients (27%) at the RD in Group A and 6 of 10 patients (60%) in Group B showed greater antitumor activity with paclitaxel and lurbinectedin with or without BEV compared to the last prior therapy (Fig. 2). The tumor types of these 15 patients were NSCLC (n = 6, all in Group B), breast, SCLC (n = 3 each), epithelial ovarian (n = 2), and endometrial cancer (n = 1).

Antitumor activity of paclitaxel and lurbinectedin with or without BEV was also observed among evaluable patients pretreated with taxanes (n = 22 at the RD in Group A, n = 7 in Group B). Among patients pretreated with taxanes, 8 confirmed responses and 5 SD ≥ 3 months occurred at the RD in Group A (ORR = 36%, 95%CI, 17.2–59.3%; CBR = 59%, 95%CI, 36.4–79.3%), and 5 confirmed responses and one SD ≥ 3 months in Group B (ORR = 71%; 95%CI, 29.0–96.3%; CBR = 86%; 95%CI, 42.1–99.6%).

Clinical benefit for > 12 months was observed in 5 patients: 4 in Group A (3 at the RD) and one in Group B. These patients had SCLC (n = 2), epithelial ovarian, endometrial cancer, and NSCLC (n = 1 each), had received 1–3 prior chemotherapy lines, and were given 18–35 cycles of study treatment each (all with PR as best response). Four of these patients showed no signs of disease progression prior to study termination.

### Pharmacokinetics

All patients were sampled for PK analysis and were suitable for non-compartmental analysis (NCA). Parameters obtained for paclitaxel and lurbinectedin at each dose level are shown in Supplemental Information. Wide variability was observed in lurbinectedin and paclitaxel total clearance (CL). No dose linearity could be established for lurbinectedin maximum concentration (C_max_) and area under the concentration–time curve (AUC). Potential drug-drug interactions (DDIs) between paclitaxel and lurbinectedin could not be fully ruled out, since a slight decrease in the CL of each drug was observed at the high AUC of the other drug. No statistically significant differences were observed in the PK parameters of paclitaxel and lurbinectedin in the presence or absence of BEV. Additional details are provided in Supplemental Information.

## Discussion

This clinical trial defined the RD for phase II studies of paclitaxel plus lurbinectedin combination at paclitaxel 80 mg/m^2^ on D1 and D8, and lurbinectedin 2.2 mg/m^2^ on D1, q3wk.

The safety profile of the paclitaxel plus lurbinectedin combination was predictable. Myelotoxicity at the RD was common but reversible and manageable, and most non-hematological toxicities were mild/moderate. As expected, severe myelotoxicity and some non-hematological toxicities (vomiting, and especially neuropathy and alopecia) were slightly more frequent at this RD compared to the established dose for lurbinectedin monotherapy (3.2 mg/m^2^ q3wk) [16, 17]. This may be attributed to the addition of paclitaxel, as these toxicities are commonly reported with single-agent paclitaxel [18–20]. No patients treated with the combination discontinued treatment due to toxicity, thereby further suggesting an acceptable safety profile.

As expected, the addition of BEV 15 mg/kg q3wk to this RD increased the incidence of some treatment-related events (e.g., hypertension, gastrointestinal events, embolism and febrile neutropenia), and severe neutropenia. Similar increases have been reported in clinical trials comparing chemotherapy plus BEV *vs.* chemotherapy alone in patients with solid tumors [8, 11, 21, 22]. Despite this additional toxicity, which was manageable, the triple combination of paclitaxel, lurbinectedin and BEV was generally well tolerated.

Encouraging antitumor activity was observed herein with the paclitaxel plus lurbinectedin combination in several indications. Recognizing the limitations of cross trial comparisons, the response rates at the RD without BEV were higher than those reported for lurbinectedin monotherapy in second-line treatment of patients with SCLC (80% *vs.* 35%) [16], *BRCA*-unselected breast cancer (50% *vs.* 9%) [23], and ovarian cancer (33% *vs.* 14–23%) [17, 24]. With the limitation of the small number of patients with each tumor type treated in this study, administration of paclitaxel plus lurbinectedin at the RD without BEV resulted in higher response rates in relapsed/refractory SCLC (80% *vs.* 20–27%) [25] and metastatic breast cancer (50% *vs.* 22–42%) [26, 27], and a similar response rate in relapsed ovarian cancer (33% *vs.* 21–60%) [20], compared with single-agent weekly paclitaxel at the dose commonly used in clinical practice (i.e., 80 mg/m^2^/week). These effects were achieved at a lower paclitaxel dose intensity (50.3 mg/m^2^/week *vs.* 72–78 mg/m^2^/week) [20] and were associated with a lower incidence of peripheral neuropathy (all grades, 27% *vs.* 50–100%; grade 3/4, 0% *vs.* 3–21%) [25, 27–31]. Finally, in patients with endometrial cancer, the response rate of 27% achieved at the RD without BEV compares favorably with the modest response rates (≤ 15%) reported in trials evaluating second-line chemotherapies in this indication, with the highest ones being found with ifosfamide [32] and ixabepilone [33, 34].

The addition of BEV improved response rate to the combination at the RD in patients with epithelial ovarian cancer (from 33 to 75%) and NSCLC (from 25 to 33%). This is in line with the finding, in previous studies, of a 15–20% improvement in ORR with the addition of BEV to chemotherapy *vs.* chemotherapy alone in patients with recurrent ovarian cancer [12, 22, 35] and previously untreated NSCLC [9, 36, 37].

The PK parameters of paclitaxel and lurbinectedin in the present study were generally similar to those reported elsewhere [6, 38]. The absence of linearity found for lurbinectedin C_max_ and AUC was probably due to the dose levels explored being very close. DDIs between paclitaxel and lurbinectedin could not be ruled out, although their clinical relevance would be marginal, based on the slight changes in CL and the large variability of PK parameters observed. BEV had no significantly effects on the PK profile of either lurbinectedin or paclitaxel.

In conclusion, weekly paclitaxel 80 mg/m^2^ on D1 and D8 combined with lurbinectedin 2.2 mg/m^2^ on D1 q3wk, with or without the addition of BEV 15 mg/kg on D1, showed a manageable overall safety profile and promising antitumor activity in patients with selected advanced solid tumors. These results support further development of this combination without BEV in the treatment of SCLC, breast, and endometrial cancer, and with added BEV in the treatment of epithelial ovarian cancer.

## Supplementary Information

Below is the link to the electronic supplementary material.

Supplementary file1 (DOCX 163 KB)

## Data Availability

Individual participant data are not publicly available since this requirement was not anticipated in the study protocol considering that this trial started patient enrolment in 2013. Clinical trial summary results were placed at ClinicalTrials.gov (https://www.clinicaltrials.gov).
