# Supplementary material for: Phase I study of lurbinectedin in combination with weekly paclitaxel with or without bevacizumab in patients with advanced solid tumors
Source: Invest New Drugs. 2022 Aug 10;40(6):1263–73. doi: 10.1007/s10637-022-01281-z (PMC9652263; doi:10.1007/s10637-022-01281-z)
Supplement: Supplementary file 1 — Supplementary file1 (DOCX 163 KB) [file 10637_2022_1281_MOESM1_ESM.docx]

Supplementary information

**Article title:** Phase I Study of Lurbinectedin in Combination with Weekly Paclitaxel with or without Bevacizumab in Patients with Advanced Solid Tumors.

**Journal name:** Investigational New Drugs.

**Author names:** Emiliano Calvo^1^, Cristiana Sessa^2^, Guilherme Harada^3^, Maria de Miguel^1^, Carmen Kahatt^4^, Xarles Erik Luepke-Estefan^4^, Mariano Siguero^4^, Carlos Fernandez-Teruel^4^, Martin Cullell-Young^4^, Anastasios Stathis^2^, and Alexander Drilon^3^.

**Author affiliation:** 1. START Madrid - HM CIOCC, Hospital Madrid Norte Sanchinarro, Madrid, Spain.

2. Oncology Institute of Southern Switzerland, EOC, Ospedale San Giovanni, Bellinzona, Switzerland.

3. Memorial Sloan Kettering Cancer Center, New York NY, U.S.A.

4. PharmaMar, Colmenar Viejo, Madrid, Spain.

**Corresponding author:** Alexander Drilon, M.D.

E-mail: [drilona@mskcc.org](mailto:drilona@mskcc.org)

Supplementary Information

Patients and Methods

*Study design*

This open-label, dose-ranging, uncontrolled phase I study was designed to determine the maximum tolerated dose (MTD) and the recommended dose (RD) of weekly paclitaxel as a 1-hour intravenous (i.v.) infusion on Day (D) 1, D8 and D15 followed by lurbinectedin as a 1-hour i.v. infusion on D1, both every three weeks (q3wk) in adult patients with selected advanced solid tumors (Group A). Once a RD had been defined for the paclitaxel and lurbinectedin combination, the feasibility of adding bevacizumab (BEV) on D1 q3wk to this regimen was explored in a selected cohort of patients (Group B). Secondary objectives comprised to characterize the safety profile and feasibility of the combination, to obtain preliminary information on its antitumor activity, and to characterize its pharmacokinetic (PK) profile and detect any major drug-drug interactions.

*Eligibility Criteria*

Eligible patients were aged 18-75 years; with advanced and/or unresectable solid tumors; life expectancy ≥3 months; who had recovered from previous toxicities; with Eastern Cooperative Oncology Group performance status score ≤1; and adequate bone marrow, hepatic, renal and metabolic function. Patients in the expansion cohort at the RD in Group A (paclitaxel and lurbinectedin), and in Group B (paclitaxel, lurbinectedin and BEV), also had measurable disease according to Response Evaluation Criteria In Solid Tumors (RECIST) v.1.1, and documented disease progression.

Patients were excluded if they had been pretreated with lurbinectedin, weekly paclitaxel-based therapy or nanoalbumin bound-paclitaxel; had received >3 lines of prior chemotherapy for advanced disease, taxane-containing therapy ≤3 months before inclusion, external pelvic irradiation >45 grays, anticoagulation with coumarin derivatives, or bone marrow and/or stem cell transplantation; had discontinued previous paclitaxel-based therapy due to toxicity; were lactating women or were not using effective contraceptives; or had symptomatic brain metastases or leptomeningeal disease, bone marrow involvement, active infection, relevant cardiac disease, any disease interfering with study outcome, or hypersensitivity to paclitaxel, BEV or any formulation component.

*Dose Escalation and Dose-limiting Toxicities*

Patient accrual began in Group A, with dose escalation following a standard 3+3 design. The starting paclitaxel dose (60 mg/m^2^) was 75% of the single-agent dose of 80 mg/m^2^ commonly used for weekly paclitaxel in clinical practice; the starting lurbinectedin dose (3.0 mg flat dose [FD]) was 43% of the RD of 7.0 mg FD determined for single-agent lurbinectedin as a 1-hour i.v. infusion on D1 q3wk [1]. During dose escalation in Group A, lurbinectedin was converted to a body surface area (BSA)-based dose (calculated by dividing the allocated FD by a BSA of 1.8 m^2^). This followed the finding, in an exploratory analysis of pooled phase II data with single-agent lurbinectedin, of a greater probability of grade 3/4 thrombocytopenia among patients with the lowest BSA values. In Group B, patients received the RD defined in Group A supplemented with BEV 15 mg/kg; a dose commonly used when adding BEV to chemotherapy regimens [2]. In both groups, paclitaxel was discontinued after Cycle 6 (i.e., 18 weeks of treatment) and patients then received lurbinectedin at the initial dose alone (Group A) or supplemented with BEV 15 mg/kg (Group B). The triple combination in Group B would be considered feasible if less than one third of treated patients had dose-limiting-toxicities (DLTs) during Cycle 1 and no treatment-associated mortality occurred.

The following DLTs were defined: grade 4 neutropenia >3 days; febrile neutropenia or neutropenic sepsis; grade 4 thrombocytopenia (or grade 3 requiring transfusion); grade 4 transaminase increase (or grade 3 for >7 days, or any grade causing dose omission or cycle delay >72 hours); grade ≥2 transaminase increase with total bilirubin increase ≥2 x upper limit of normal (ULN) and normal alkaline phosphatase (AP); any clinically relevant grade ≥3 toxicity; cycle delay >2 weeks; and omission of two paclitaxel doses (or one paclitaxel dose and subsequent cycle delay) due to toxicity. Grade 3 neutropenia and/or thrombocytopenia ≥1 week were also defined as DLTs for cohorts without paclitaxel D15 administration.

*Study Treatment*

Lurbinectedin was supplied as a lyophilized powder concentrate, reconstituted, and diluted with glucose 5% or sodium chloride 0.9% solution. Commercially available paclitaxel and BEV were provided. Antiemetic prophylaxis was given before each paclitaxel infusion.

Patients could receive granulocyte colony-stimulating factor (G-CSF) support for febrile neutropenia or neutropenic infection at any time during the study. Primary prophylaxis with G-CSF was not allowed.

*Safety and Efficacy Assessments*

Patients were evaluable for the determination of the MTD and the RD if they received at least one lurbinectedin infusion, two complete paclitaxel infusions and, if applicable, one complete BEV infusion, except if early discontinuations or missed doses and/or assessments were the consequence of drug-related toxicity (excluding severe hypersensitivity reaction).

Patients were evaluable for safety if they received at least one partial infusion of lurbinectedin. Hematology and biochemistry tests were done at baseline, weekly during Cycle 1, on D1 and D8 (and D15 for cohorts with paclitaxel D15 administration) during Cycles 2-6, and on D1 during subsequent cycles. Electrocardiograms were done at baseline, and repeated if clinically indicated.

Patients were evaluable for efficacy if they received at least one lurbinectedin infusion and had at least one assessment as per RECIST v.1.1; if they were considered to have treatment failure (defined as symptomatic deterioration or death due to progression); or if treatment was discontinued due to any treatment-related toxicity. Efficacy endpoints comprised overall response rate (ORR, percentage of patients with complete [CR] or partial response [PR]), clinical benefit rate (CBR, percentage of patients with CR, PR or stable disease [SD] for ≥3 months, and time-to-event parameters (progression-free survival [PFS] and duration of response [DoR]).

*Pharmacokinetic Assessments*

Blood samples for PK analysis were taken at baseline, at 5 min before end of infusion (EOI) of paclitaxel, at 5 min before EOI of lurbinectedin, and at different times (1, 2, 3, 4, 5, 22, 46, 94 and 166 hours) after EOI of lurbinectedin. Both drugs were measured by validated liquid extraction methods followed by ultra-performance liquid chromatography tandem mass-spectrometry detection (Dynakin, Derio, Spain). The calibration range was 0.1-50 ng/mL for lurbinectedin and 5-1000 ng/mL for paclitaxel. Samples higher than 50 ng/mL (lurbinectedin) and 1000 ng/mL (paclitaxel) were 20x diluted following a validated procedure.

Results

*Treatment Administration*

Table S1 shows the DLTs reported during dose escalation in the 52 evaluable patients treated with lurbinectedin and paclitaxel at five dose levels in Group A, and in the 12 evaluable patients treated with lurbinectedin, paclitaxel and BEV in Group B.

**Table S1.** Distribution of patients and dose-limiting toxicities over the dose levels studied.

| **Dose level** | **Dose** | | | **No. of patients with DLTs / No. of evaluable patients** | | **Description of DLTs** |
| --- | --- | --- | --- | --- | --- | --- |
|  | **Paclitaxel**  **(mg/m^2^)** | **Lurbinectedin**  **(mg FD)** | **BEV**  **(mg/kg)** |  |  |  |
| **Group A (paclitaxel / lurbinectedin)** | | | | | | |
| **DL1** | 60 ^a^ | 3.0 | . | 0 / 3 | | . |
| **DL2** | 60 ^a^ | 4.0 | . | 0 / 3 | | . |
| **DL3** | 60 ^a^ | 5.0 | . | **2** / 6 | | Grade 4 neutropenia for >3 days (n=2) ^b^ |
| **DL4**  **(MTD)** | 80 ^c^ | 5.0 | . | **3** / 6 | | Grade 3/4 neutropenia for >7 days (n=1) |
|  |  |  |  |  |  | Grade 3 neutropenia for >7 days (n=1) ^b^ |
|  |  |  |  |  |  | Lack of compliance due to neutropenia (n=1) ^b^ |
| **DL5**  **(RD)** | 80 ^c^ | 4.0 | . | Overall:  **6** / 34 | 0 / 6 | . |
|  |  | Cohort expansion:  2.2 mg/m^2^ |  |  | Cohort expansion:  **6** / 28 | Grade 3 neutropenia for >7 days (n=2) |
|  |  |  |  |  |  | Grade 4 neutropenia for >3 days (n=1) |
|  |  |  |  |  |  | Grade 3 neutropenia for >7 days and grade 3 anemia (n=1) ^b^ |
|  |  |  |  |  |  | Grade 2 anemia and grade 2 tooth infection (n=1) ^b^ |
|  |  |  |  |  |  | Grade 3 vomiting (n=1) |
| **Group B (paclitaxel / lurbinectedin / BEV)** | | | | | | |
| **RD + BEV** | 80 ^c^ | 2.2 mg/m^2^ | 15 | **3** / 12 | | Grade 4 large intestine perforation (n=1) |
|  |  |  |  |  |  | Grade 3 febrile neutropenia (n=1) |
|  |  |  |  |  |  | Grade 4 neutropenia for >3 days (n=1) |
| Three patients treated at the RD in Group A and two patients treated in Group B were not evaluable for DLTs.  ^a^ On D1, D8 and D15 q3wk.  ^b^ Delayed DLTs, i.e. that occurred after Cycle 1.  ^c^ On D1 and D8 q3wk.  BEV, bevacizumab; D, day; DL, dose level; DLT, dose-limiting toxicity; FD, flat dose; MTD, maximum tolerated dose; q3wk, every three weeks; RD, recommended dose. | | | | | | |

*Pharmacokinetics*

Table S2 summarizes PK parameters of paclitaxel and lurbinectedin in Cycle 1 for each of the five dose levels in Group A, and also for Group B.

**Table S2.** Non-compartmental pharmacokinetic parameters of paclitaxel and lurbinectedin by dose level.

|  | **Group A** | | | | | |  | **Group B** |
| --- | --- | --- | --- | --- | --- | --- | --- | --- |
|  | **Paclitaxel (mg/m^2^)**  **D1,D8,D15**  **/ lurbinectedin (mg FD)** | | | **Paclitaxel (mg/m^2^)**  **D1,D8**  **/ lurbinectedin (mg FD)** | | |  | **Paclitaxel (mg/m^2^) D1,D8**  **/ lurbinectedin (mg/m^2^)**  **/ BEV (mg/kg)** |
|  | **DL1**  60 / 3.0 | **DL2**  60 / 4.0 | **DL3** 60 / 5.0 | **DL4 (MTD)** 80 / 5.0 | **DL5 (RD)** | |  | **RD + BEV**  80 / 2.2 / 15 |
|  |  |  |  |  | 80 / 4.0 | 80 / 2.2 mg/m^2^ |  |  |
| **Paclitaxel** | *n = 3* | *n = 3* | *n = 6* | *n = 6* | *n = 6* | *n = 31* |  | *n = 12* |
| AUC (h* μg/L) | 4071  (835.8) | 4523  (2874) | 3508  (1163) | 4830  (1224) | 4998  (759.8) | 4861  (1257) |  | 4163  (1265) |
| CL (L/h) | 26.2  (9.0) | 29.2  (15.1) | 34.5  (16.1) | 30.2  (8.6) | 31.1  (6.2) | 31.5  (9.6) |  | 36.9  (11.1) |
| C_max_ (μg/L) | 2370  (173.5) | 1497  (834.9) | 1982  (756.6) | 3122  (1420) | 2908  (482.2) | 3004  (971.4) |  | 2567  (1077) |
| HL (h) | 18.2  (0.8) | 29.2  (22.6) | 16.5  (2.8) | 14.4  (1.3) | 14.3  (1.1) | 14.3  (1.9) |  | 15.3  (1.5) |
| V_ss_ (L) | 314.7  (99.0) | 577.4  (286.7) | 429.9  (275.2) | 323.2  (266.8) | 315.3  (89.7) | 324.4  (219.3) |  | 386.0  (147.5) |
| V_z_ (L) | 689.2  (241.7) | 934.4  (216.8) | 858.3  (568.2) | 627.5  (185.2) | 639.9  (128.6) | 662.4  (274.0) |  | 807.9  (223.2) |
| **Lurbinectedin** | *n = 3* | *n = 3* | *n = 6* | *n = 6* | *n = 6* | *n = 31* |  | *n = 12* |
| AUC (h* μg/L) | 405.9 (129.6) | 357.9  (174.2) | 714.0  (416.3) | 376.2  (151.4) | 436.2  (238.3) | 440.5  (224.4) |  | 351.2  (175.9) |
| CL (L/h) | 7.8  (2.2) | 14.2  (9.4) | 8.8  (4.0) | 15.2  (6.0) | 12.0  (7.0) | 11.1  (5.3) |  | 13.8  (6.5) |
| C_max_ (μg/L) | 97.7  (45.5) | 74.0  (18.9) | 127.0  (74.0) | 109.5  (28.4) | 83.9  (42.5) | 96.3  (34.7) |  | 88.7  (28.9) |
| HL (h) | 52.8  (3.7) | 82.1  (65.7) | 46.6  (15.5) | 38.6  (8.3) | 47.1  (23.0) | 49.3  (22.5) |  | 40.0  (15.7) |
| V_ss_ (L) | 299.4 (84.4) | 733.7  (415.1) | 343.2  (150.1) | 401.8  (99.1) | 420.9  (182.7) | 454.9  (222.6) |  | 408.5  (161.7) |
| V_z_ (L) | 602.1 (193.0) | 1161  (563.2) | 530.8  (164.6) | 806.0  (243.4) | 701.8 (268.9) | 727.6  (346.9) |  | 724.9  (340.5) |
| Values are expressed as mean (standard deviation).  AUC, area under the concentration-time curve from time zero to infinity; BEV, bevacizumab; CL, total clearance; C_max_, maximum concentration; D, Day; DL, dose level; HL, terminal half-life; MTD, maximum tolerated dose; RD, recommended dose; V_ss_, volume of distribution at steady-state; V_z_, apparent volume of distribution during terminal phase. | | | | | | | | |

The paclitaxel and lurbinectedin PK parameters transformed into the natural logarithm) obtained in Cycle 1 at the RD in Group A and in Group B (paclitaxel plus PM01183 and BEV) were compared to assess the effect of BEV. No statistically significant differences were found for paclitaxel (Table S3) or lurbinectedin (Table S4) parameters, therefore suggesting that BEV has no effects on the PK profile of either drug.

**Table S3.** Differences in paclitaxel pharmacokinetic parameters between groups.

| **Parameter** | **Group** | **No. of patients** | **Minimum** | **Maximum** | **Mean** | **SDev** | **p value** |
| --- | --- | --- | --- | --- | --- | --- | --- |
| AUC | A | 31 | 7.825 | 8.991 | 8.456 | 0.264 | 0.0864 |
|  | B | 12 | 7.812 | 8.790 | 8.292 | 0.303 | . |
| CL | A | 31 | 2.928 | 4.094 | 3.410 | 0.291 | 0.1320 |
|  | B | 12 | 2.985 | 4.058 | 3.565 | 0.312 | . |
| C_max_ | A | 31 | 6.477 | 8.364 | 7.933 | 0.442 | 0.2177 |
|  | B | 12 | 6.692 | 8.425 | 7.752 | 0.494 | . |
| HL | A | 31 | 2.280 | 2.968 | 2.650 | 0.132 | 0.0694 |
|  | B | 12 | 2.563 | 2.891 | 2.724 | 0.097 | . |
| AUC, area under the concentration-time curve from time zero to infinity; CL, total body clearance; C_max_, maximum plasma concentration; HL, terminal half-life; PK, pharmacokinetic; SDev, standard deviation. | | | | | | | |

**Table S4.** Differences in lurbinectedin pharmacokinetic parameters between groups.

| **Parameter** | **Group** | **No. of patients** | **Minimum** | **Maximum** | **Mean** | **SDev** | **p value** |
| --- | --- | --- | --- | --- | --- | --- | --- |
| AUC | A | 31 | 5.065 | 7.038 | 5.972 | 0.488 | 0.1751 |
|  | B | 12 | 4.949 | 6.535 | 5.746 | 0.505 | . |
| CL | A | 31 | 1.305 | 3.219 | 2.296 | 0.474 | 0.1596 |
|  | B | 12 | 1.808 | 3.175 | 2.519 | 0.487 | . |
| C_max_ | A | 31 | 3.918 | 5.236 | 4.513 | 0.329 | 0.4266 |
|  | B | 12 | 3.850 | 4.949 | 4.432 | 0.352 | . |
| HL | A | 31 | 2.720 | 4.736 | 3.802 | 0.447 | 0.2013 |
|  | B | 12 | 2.719 | 4.140 | 3.603 | 0.459 | . |
| AUC, area under the concentration-time curve from time zero to infinity; CL, total body clearance; C_max_, maximum plasma concentration; HL, terminal half-life; PK, pharmacokinetic; SDev, standard deviation. | | | | | | | |

In an additional analysis of potential drug-drug interactions, the AUC of lurbinectedin was compared against the PK parameters of paclitaxel, and the AUC of paclitaxel was compared against the PK parameters of lurbinectedin. A slight interaction was detected, where the CL for both drugs decreased depending on the achieved AUC of the other drug (Figure S1).

**Figure S1.** Total clearance profiles for paclitaxel *vs.* area under the concentration-time curve for lurbinectedin (A), and for lurbinectedin *vs.* area under the concentration-time curve for paclitaxel (B).

**
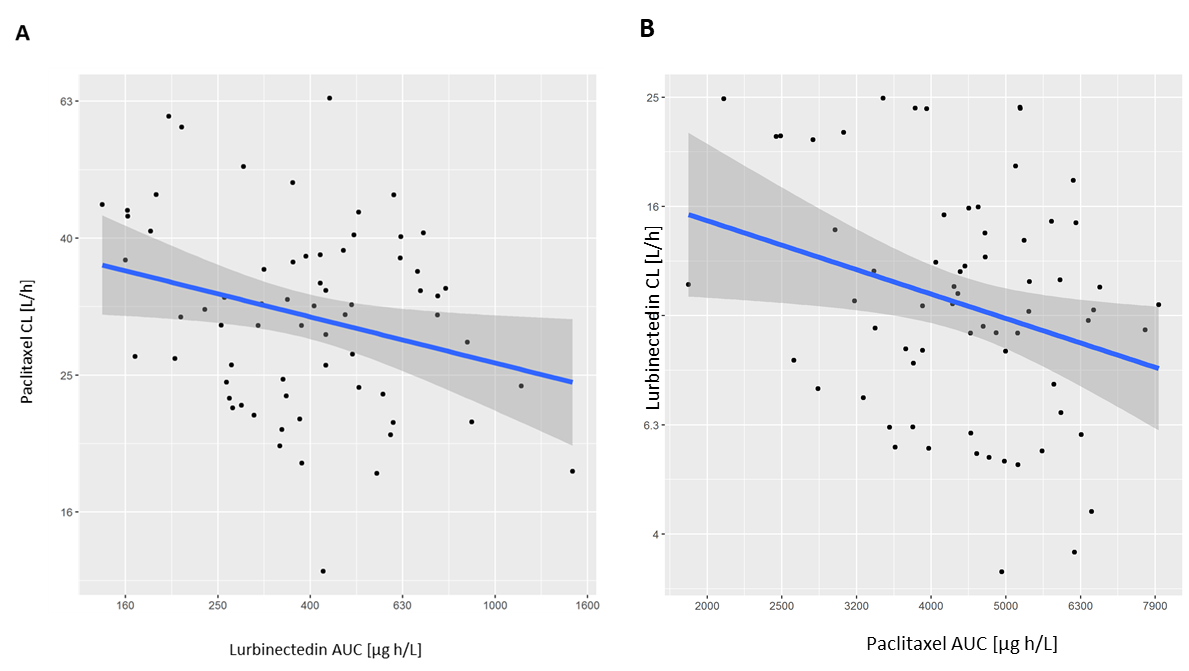
**

AUC, area under the concentration-time curve; CL, total clearance.

References

1. Elez ME, Tabernero J, Geary D, Macarulla T, Kang SP, Kahatt C, Pita AS, Teruel CF, Siguero M, Cullell-Young M, Szyldergemajn S and Ratain MJ (2014) First-in-human phase I study of Lurbinectedin (PM01183) in patients with advanced solid tumors. Clin Cancer Res 20(8):2205-14.

2. Lima AB, Macedo LT and Sasse AD (2011) Addition of bevacizumab to chemotherapy in advanced non-small cell lung cancer: a systematic review and meta-analysis. PLoS One 6(8):e22681.
